# Human resource management research in healthcare: a big data bibliometric study

**DOI:** 10.1186/s12960-023-00865-x

**Published:** 2023-12-05

**Authors:** Xiaoping Qin, Yu-Ni Huang, Zhiyuan Hu, Kaiyan Chen, Lin Li, Richard Szewei Wang, Bing-Long Wang

**Affiliations:** 1 School of Health Policy and Management, Chinese Academy of Medical Sciences and Peking Union Medical College, Beijing, 100730 China; 2 College of Medical and Health Science, Asia University, Taichung, 41354 Taiwan; 3 Department of Education, Peking Union Medical College Hospital, Chinese Academy of Medical Sciences & Peking Union Medical College, Beijing, 100730 China; 4 Department of Innovative Medical Research, Hospital Management Institute, Chinese People’s Liberation Army General Hospital, Beijing, 100853 China; 5 Affiliation Program of Data Analytics and Business Computing, Stern School of Business, New York University, New York, 10012 United States of America; 6 Tsinghua-Berkeley Shenzhen Institute, Tsinghua University, Shenzhen, 518055 China

**Keywords:** Human Resource Management, HRM, Big Data, Bibliographic analysis, Health Trends, Healthcare

## Abstract

Human resource management (HRM) in healthcare is an important component in relation to the quality and efficiency of healthcare delivery. However, a comprehensive overview is lacking to assess and track the current status and trends of HRM research in healthcare. This study aims to describe the current situation and global trends in HRM research in healthcare as well as to indicate the frontiers and future directions of research. The research methodology is based on bibliometric mapping using scientific visualization software (VOSviewer). The data were collected from the Web of Science(WoS) core citation database. After applying the search criteria, we retrieved 833 publications, which have steadily increased over the last 30 years. In addition, 93 countries and regions have published relevant research. The United States and Australia have made significant contributions in this area. Current research articles focus on topics clustered into performance, hospital/COVID-19, job satisfaction, human resource management, occupational/mental health, and quality of care. The most frequently co-occurring keywords are human resource management, job satisfaction, nurses, hospitals, health services, quality of care, COVID-19, and nursing. There is limited research on compensation management and employee relations management, so the current HRM research field still has not been able to present a complete and systematic roadmap. We propose that our colleagues should consider focusing on these research gaps in the future.

## Introduction

Among the many management elements, people are the most dynamic and active element, and they are an important asset in organizations [1]. The term “human resources” was first coined by the academic Peter F. Drucker in 1954 [2]. The key function of human resources management (HRM) is to “put the right people in the right jobs at the right time” [2]. HRM refers to the planned allocation of human resources in accordance with the requirements of organizational development through a series of processes, such as recruitment, training, use, assessment, motivation, and adjustment of employees, to mobilize their motivation, bring into play their potential and create value for the organization [1]. Ensuring the achievement of the organization’s strategic objectives, HRM activities mainly include human resource strategy formulation, staff recruitment and selection, training and development, performance management, compensation management, staff mobility management, staff relationship management, staff safety and health management, etc. Similarly, modern healthcare management has human resources as the core. The HRM level in hospitals is related to the quality and efficiency of medical services provided by hospitals, which is also the core of internal hospital management and the focus of health macro management [3].

The World Health Organization (WHO) states that health systems can only work with the help of health workers, and that improving the coverage of health services and realizing the right to the highest standard of health depends on the availability, accessibility, acceptability and quality of health workers [4]. In response to evolving characteristics in socio-economic development and the human resource market, healthcare system personnel reforms are evident in three key areas: first, decentralization and flexible employment practices grant hospital managers greater decision-making autonomy concerning priorities and access to medical resources. However, they also impose quantitative and functional constraints on physicians' working hours, career planning, and medical payment systems. Second, a focal point is the rational allocation of technical staff to achieve efficiency while controlling labor costs. Finally, hospital organization change and restructuring are prevalent. Many European countries have unionized hospital employees, limiting the ability to establish independent incentives and rewards. In contrast, U.S. hospital employees often do not belong to specific organizations, leading cost control efforts to revolve around adjusting the allocation of technical staff and employee numbers to reduce labor expenses [5–7].

The current global trend in the number of publications on HRM in healthcare is rising. However, there are currently several problems in HRM research. The following issues mainly exist: (1) the expertise and professionalism of HRM managers are limited. (2) Theoretical methods and technical applications are weak. (3) Insufficient regulation of regulations, systems and procedures. (4) Management is mainly at the level of operational work, and functions are too fragmented [8, 9]. Although hospitals worldwide generally recognize the importance of HRM, they do not pay sufficient attention to it. The management of human resources is also stuck in the previous understanding that its work is carried out only by transferring positions in hospitals, promoting and reducing the salary of employees and a series of other operations [10]. Most senior management in hospitals have comprehensive medical knowledge; some are experts in a particular field. Still, they lack expertise in HRM, which makes them work in a transactional way in HRM. There is also currently a general health workforce imbalance in countries worldwide. The lack of well-being of healthcare workers is particularly problematic in foreign healthcare institutions [11], and to reduce costs, some organizations have reduced staffing levels. In turn, because of lower quality of service, the morale of healthcare providers often suffers. Patient satisfaction may decline [12]. In the process of data gathering, we found that the literature related to HRM in healthcare is still under-reported and that the research topics are scattered, and there is still a lack of generalization and summary of these literatures [13]. There is no systematic theoretical support in the current research, which defines the perspective that researchers should take when analyzing and interpreting the data to be collected, leading to biased interpretations of the results, and does not allow other researchers to combine the findings with existing research knowledge and then apply them to practice [14]. Second, data collection was not rigorous, and the downloading strategy was not appropriate to achieve completeness and accuracy of data. There is also a lack of information and incomplete use of features in the presentation of knowledge maps and visualization results [15].

Therefore, the aims of this study are the following; first, we provide a new way of viewing the field of healthcare HRM and its associations by examining co-occurrence data. Second, we relate our evolutionary analysis to a comprehensive future research agenda which may generate a new research agenda in healthcare hospital HRM. This review, therefore, focuses on illuminating the research frontiers and future roadmap for healthcare HRM research [16, 17].

## Materials and methods

This study provides a bibliometric analysis of the HRM research literature in health care over a 30-year period to describe the landscape and trajectory of change in the research field. The methodology used for this overview is based on bibliometric mapping [18, 19], a visualization technique that quantitatively displays the landscape and dynamic aspects of the knowledge domain [20]. Data were collected from the Web of Science (WoS) core citation database. Two Java-based scientific visualization software packages (CiteSpace and VOSviewer), developed by Chaomei Chen and Van Eck and Waltman, were used to analyze the data [18, 21].

The data for this study were retrieved from the Web of Science on 28 September 2022. Web of Science was chosen as the search engine, because it is the most widely accepted and commonly used database for analyzing scientific publications [22]. The keywords “human resource management” and “healthcare organization” were used as search topics. First, to get a complete picture of HRM research, we searched all the literature from 1977 to the date of the search.

**Fig. 1.**
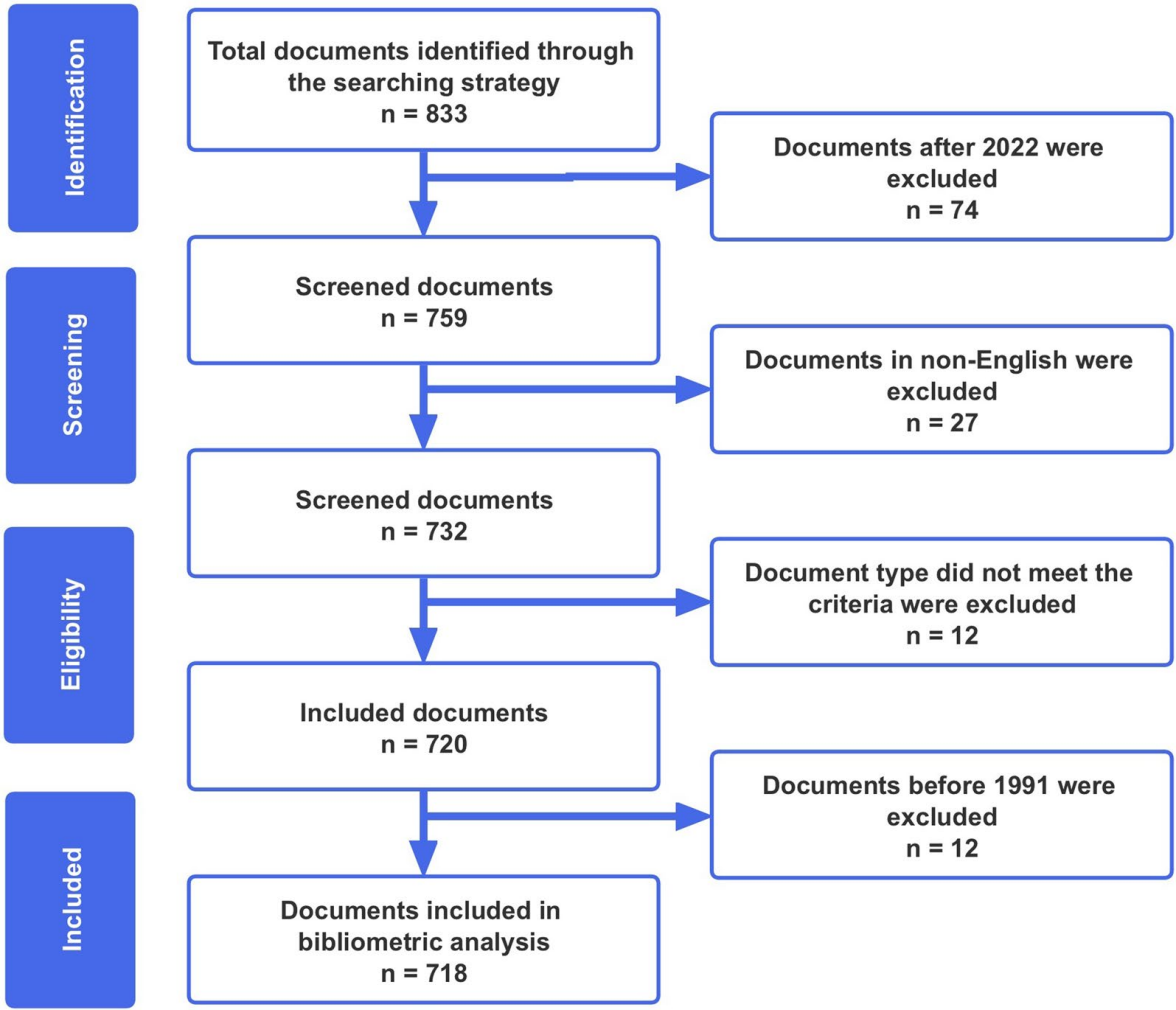
Research flow chart of the bibliometric analysis

Eight hundred thirty-three publications on HRM in healthcare organizations were identified (Fig. 1). We excluded publications before 1990, because the two documents before 1990 did not include complete information. In addition, articles, review articles, and early access articles were included in the study. To minimize language bias, we excluded literature published in languages other than English. Each publication in WoS contains detailed information, including the year of publication, author, author’s address, title, abstract, source journal, subject category, references, etc. A detailed description of the contents of the database preceded the bibliographic analysis. For example, some authors presented their names in different spellings when submitting articles, so reviewing and integrating the data in detail was necessary. A total of 718 publications were included and exported to VOSviewer and CiteSpace software to analyze the following topics: global publishing trends, countries, journals, authors, research orientations, institutions, and quality of publications.

### Introduction to CiteSpace and VOSviewer

VOSviewer is a software tool for building and visualizing bibliometric networks. It was developed by Van Eck and Waltman [21]. In VOSviewer, metric networks can be visualized and analyzed for factors, including journals, researchers, or individual publications. They can be constructed based on citations, bibliographic couplings, co-citations, or co-authorship relationships [21].

## Results

### Global publication trends

#### Number of global trends

**Fig. 2.**
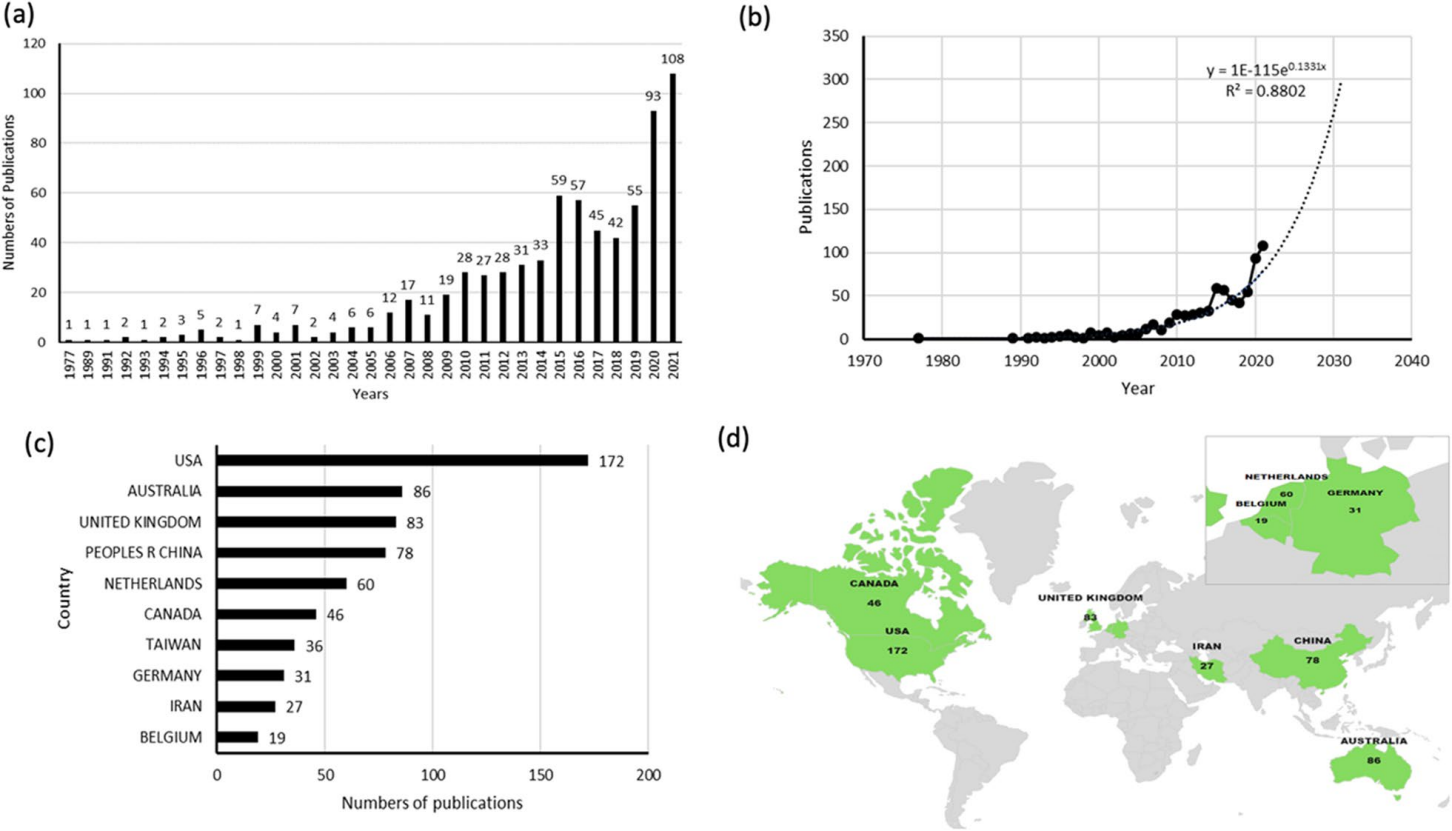
**a **Total number of publications related to HRM research. The bars indicate the number of publications per year. **b** Model fitting curves of global publication trends. **c** Top 10 countries of total publications. **d** Distribution world map of HRM research

After applying the search criteria, we retrieved a total of 718 articles. Figure 2a shows the increase in articles from 1 in 1977 to 108 in 2021. To predict future trends, a linear regression model was used to create a time curve for the number of publications throughout the year, and the model fit curve for the growth trend is shown in Fig. 2b. The trend in the number of publications fitted the time curve well at *R*^2^ = 0.8802. The R-squared value is a measure of how well the trend line fits. This value reflects the degree of fit between the estimated value of the trend line and the corresponding actual data; the better the fit, the more reliable the trend line is [23, 24]. Based on the model’s trends, it is also predicted that the number of articles on HRM in healthcare will increase to approximately 300 by 2030, an almost threefold increase compared to 2021.

#### Country and regional contributions

Figure 2c, d shows the number of publications and the world distribution of the top 10 countries in total publication numbers. The USA contributed the most publications (172, 24.2%), followed by Australia (86, 12.0%), the UK (83, 11.6%), and China (78, 10.9%).

#### Total number of citations

**Fig. 3:**
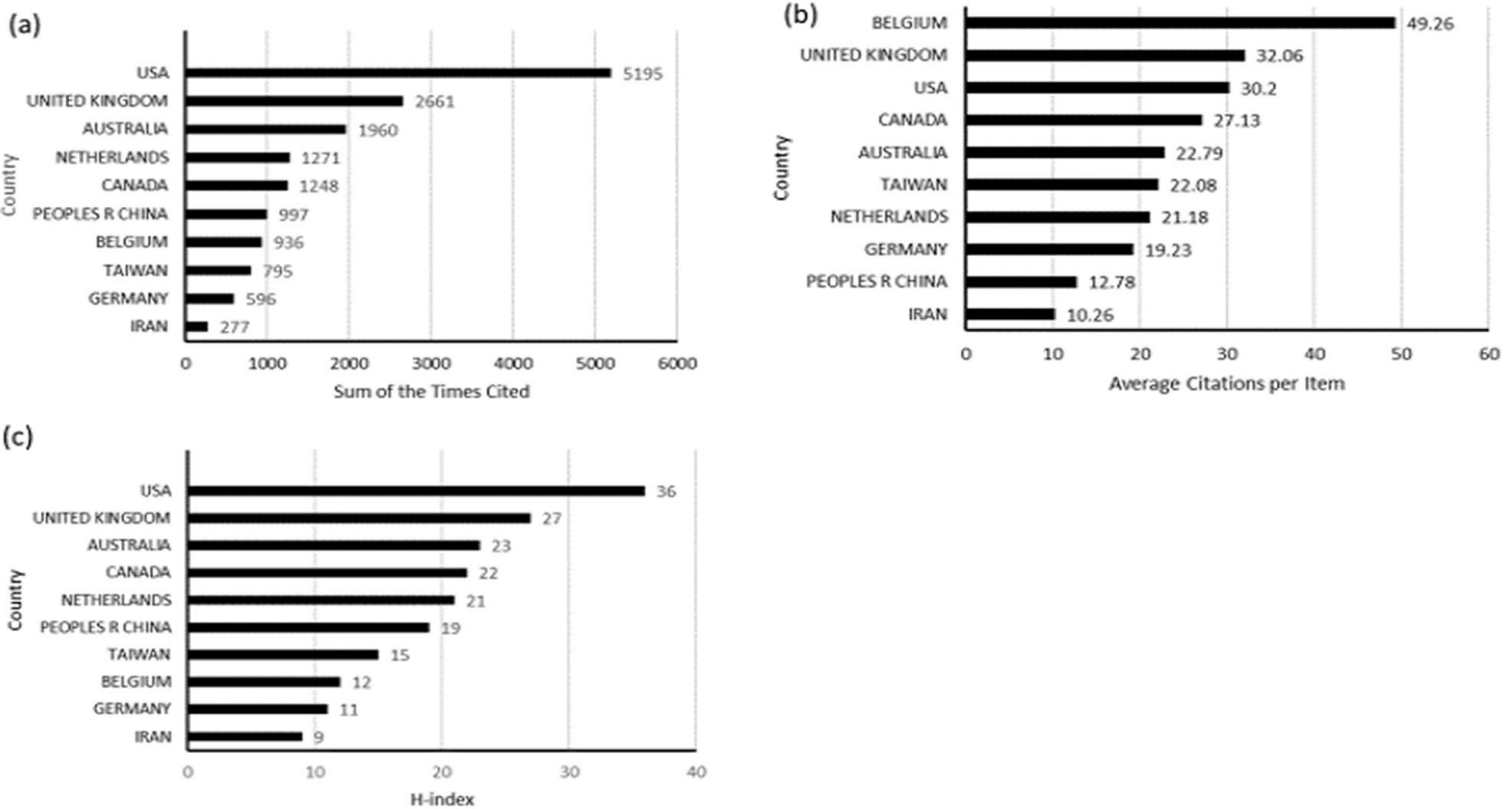
**a** Top 10 countries of average citations for each article. **b** Average number of citations. **c** Top 10 countries of the H-index

The USA had the highest total number of citations of all included publications (5195) (Table 1), while the UK ranked second (2661), followed by Australia (1960) and the Netherlands (1271). The detailed rankings and numbers are shown in Fig. 3a and Table 1.

**Table 1:** Contributions in publications of countries

Country	Publications	Sum of the Times Cited	Average Citations per Item	H-index
USA	172	5195	30.2	36
UNITED KINGDOM	83	2661	32.06	27
AUSTRALIA	86	1960	22.79	23
NETHERLANDS	60	1271	21.18	21
CANADA	46	1248	27.13	22
CHINA	78	997	12.78	19
BELGIUM	19	936	49.26	12
TAIWAN	36	795	22.08	15
GERMANY	31	596	19.23	11
IRAN	27	277	10.26	9

#### Average citation frequency

Belgium had the highest average number of citations (49.26), followed by the UK (32.06), the USA (30.2), and Canada (27.13), as shown in Fig. 3b.

#### H-index

Total citations and the h-index reflect the quality of a country’s publications and academic impact[25]. Figure 3c shows the ranking of the h-index, where the top country is the USA (h-index = 36), followed by the UK (h-index = 27), Australia (h-index = 23), and Canada (h-index = 22).

### Analysis of publications

#### Journals

Table 2 shows the top 10 journals for publications on HRM in healthcare, with 54 articles published in “International Journal of Human Resource Management”, 44 articles published in “BMJ Open”, 30 articles published in “Journal of Nursing Management”, and 24 articles in “BMC Health Services Research”.

**Table 2.** Top 10 journals of publications related to HRM research

Publications	Times	Percentage(*n* = 718)
International Journal Of Human Resource Management	54	7.521
Bmj Open	44	6.128
Journal Of Nursing Management	30	4.178
Bmc Health Services Research	24	3.343
Journal Of Advanced Nursing	18	2.507
Health Care Management Review	16	2.228
Human Resources For Health	16	2.228
Human Resource Management	14	1.95
Plos One	14	1.95
Human Resource Management Journal	11	1.532

#### Authors

Table 3 shows the top 10 most published authors with 96 articles/reviews in the last decade, representing 13.4% of all literature in the field. Timothy Bartram from Australia has published 19 papers, followed by Sandra Leggat from Australia, Stanton P from the USA, and Townsend K from the UK with 13, 11, and 10 papers, respectively. All researchers listed as authors were included in this term for analysis, regardless of their relative contribution to the study. Notably, we have included all authors in this analysis regardless of their relative contribution to the study.

**Table 3:** Top 20 authors of publications

Author	Publications	Sum of the Times Cited	Average Citations per Item	h-index
Bartram T	19	722	38	12
Leggat SG	13	488	37.54	9
Stanton P	11	510	46.36	8
Townsend K	10	210	21	8
Wilkinson A	10	210	21	8
Van Rhenen W	8	138	17.25	5
Paauwe J	7	258	36.86	4
Boselie P	6	338	56.33	6
Kellner A	6	87	14.5	6
Marchal B	6	163	27.17	6

#### Research orientation

**Fig. 4.**
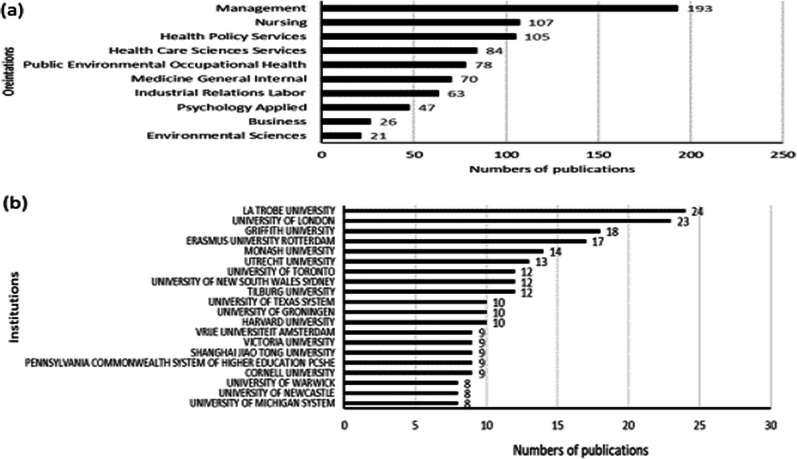
**a** Top 10 research orientations and the number of publications in each orientation. **b** Top 20 institutions with the most publications

Figure 4a shows the top 10 research orientations of the 100 research orientations. The most common research orientations were management (193 articles), nursing (107 articles), health policy services (105 articles), and health care sciences services (201 articles).

#### Institutions

Figure 4 shows the top 20 institutions with the most published papers. La Trobe University has the highest number of articles with 24, followed by the University of London (23) and Griffith University (18).

### Co-occurrence analysis

**Fig. 5.**
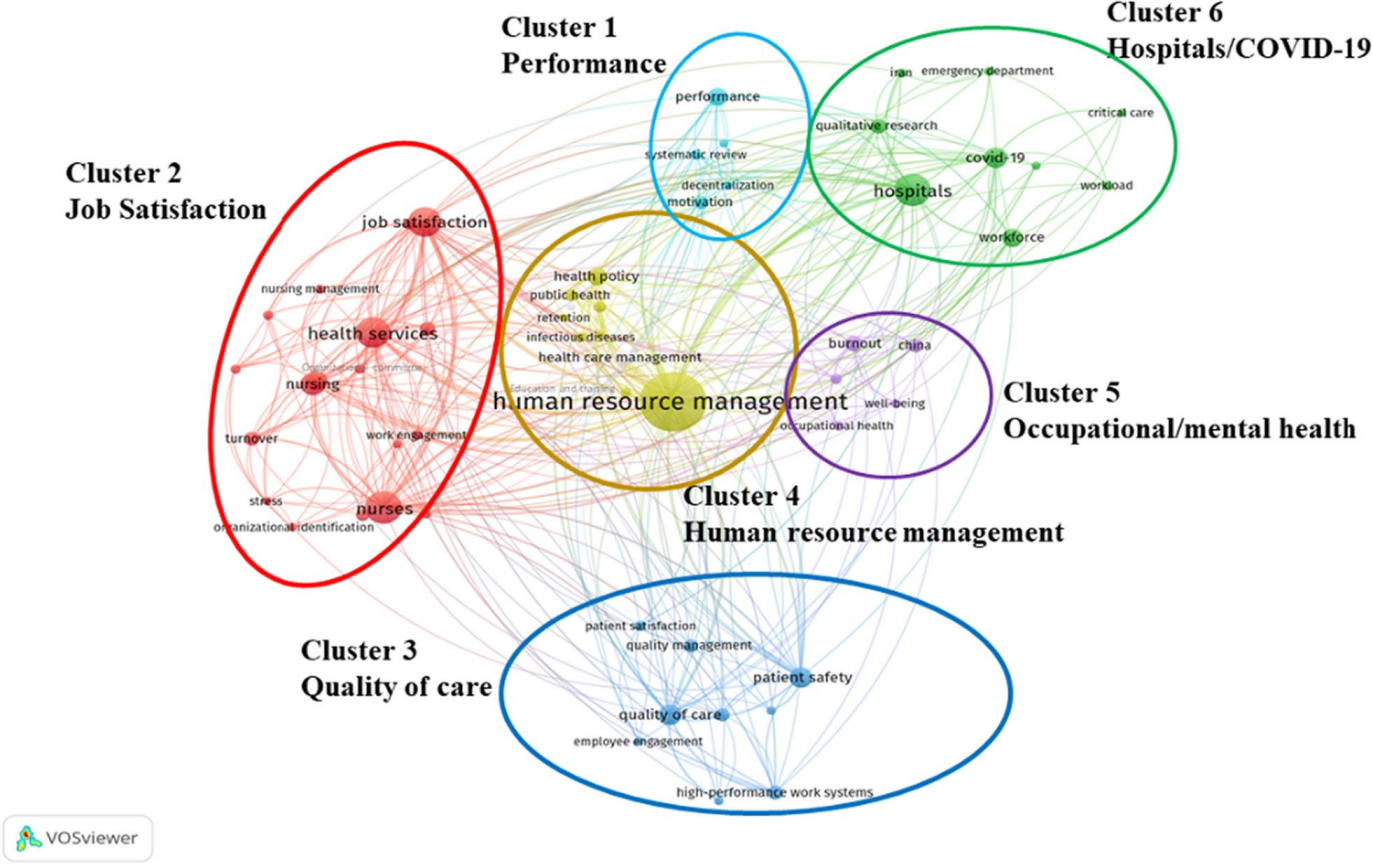
Co-occurrence analysis of HRM research in healthcare

In the keyword mapping on HRM research in healthcare, the size of the nodes represents the frequency, while the line between the nodes reflects the co-occurrence relationship. A total of 1914 keywords were included, and 59 met the criteria. All keywords were grouped into six clusters: performance (light blue cluster), job satisfaction (red cluster), quality of care (blue cluster), human resource management (brown cluster), occupational/mental health (purple cluster), and hospital/COVID-19 (green cluster) (Fig. 5).

The most prominent themes in HRM research in healthcare are as below. In the “Performance” cluster, the keywords which have the greatest co-occurrence strength were “performance”, “systematic review”, “decentralization health system” and “motivation”. The main keywords in the “Job Satisfaction” cluster are “job satisfaction”, “organizational commitment”, “transformational leadership” and “turn over”. In the “Quality of care” cluster, the keywords that stand out are “quality of care”, “patient safety”, “high-performance work system”, “quality management” and “patient satisfaction”. In the “Human resource management” cluster, the prominent keywords include “human resource management”, “health policy”, “public health”, and “education and training”. In the “Occupational/Mental Health” cluster, the prominent keywords are “Occupational health”, “mental health”, “well-being” and “burnout”. The main keywords in the “Hospital/COVID-19” cluster were “hospitals”, “COVID-19” “workforce” and “qualitative research”.

## Discussion

### Global trends in HMR in healthcare

Our study of HMR research in healthcare illustrates current and global trends in publications, contributing countries, institutions, and research orientations. The field of HMR research has evolved over the past three decades. However, as this study shows, the number of publications steadily increases yearly, with 93 countries or regions publishing in the field, suggesting that research focusing on HMR research and providing in-depth knowledge will likely increase.

### Quality and status of publications worldwide

We find that most publishing countries are developed countries, but developing countries are catching up. The total citation rate and the h-index reflect the quality and scholarly impact of a country’s publications [25]. According to our study, the US ranks first among other countries in total publications, citations, and h-index, making the most substantial contribution to global HRM research. The UK and Canada also contribute significantly, with impressive total citation frequencies and h-index, especially the UK, which ranks second in average citation frequency. However, some countries, such as Belgium, Canada and Australia, also play an important role, given their high average citation frequency. In developing countries, HRM research has also served as a guide for hospitals to improve the quality of care. The study will serve as a reference for developing countries to learn from the experience of developed countries as their economic development gradually catches up with that of developed countries.

The impact and prestige of the journals can be seen in the number of articles published in the field and the influential journals in healthcare HRM research, including the BMC Health Services Research, the Journal of Nursing Management, the International Journal of Human Resource Management, the Health Care Management Review, and the Journal of Health Organisation and Management. These high-quality journals are thus the main source of information for researchers in this field on the latest developments in HRM in healthcare.

The study shows that almost all of the top 20 institutions come from the top five countries with the most publications, with the majority coming from the US, Australia and the Netherlands, reflecting the great academic influence of these three countries in the field of HRM in healthcare. These institutions play an important role in raising the academic performance of a country. Furthermore, the top 20 authors represent research leaders who are likely to impact the future direction of research significantly. Therefore, more attention should be paid to their work to stay abreast of the latest developments in the field.

### Research Focus on HRM

Keywords play a crucial role in research papers as they contain vital information [26]. A systematic analysis of keywords within a specific research domain offers valuable insights into trends and focal points across various research areas [27]. Moreover, co-occurrence analysis relies on the number of joint publications to evaluate relationships among the identified keyword domains. As a result, it serves as an effective method for predicting future trends and focal points within the research areas of interest. These findings are expected to inspire more researchers to contribute to the future of HRM research in healthcare [28].

In this study, a total of six research domains were eventually summarized. Performance, Hospital/COVID-19, Job Satisfaction, Human resource management, Occupational/Mental Health, and Quality of care. By visualizing the analysis results, we can easily further clarify future trends. As the co-occurrence diagram shows, the keywords “Organizational culture”, “Patient safety”, “Nursing”, “Leadership”, “Quality of care” and “Hospitals” are highlighted as larger icons, so that investment and demand for quality research are necessary for the context of these six research directions.

### Six modules and research directions in human resources

This study found that the visual clustering results and the keywords that emerged from the clusters were closely related to the HRM module*s* described in “Human Resources Management: Gaining a Competitive Advantage” by Noe. R*. *[29]. The modules have been cited in HRM research and are used as textbooks in universities [30–33]. Some of the keywords in each cluster correspond to human resource planning, performance management, recruitment and staffing, and training and development, respectively. The explanation of the HRM modules is described in the next paragraph. However, there are no explicit keywords in the modules related to employee relations management and compensation management results. This may be due to the private nature of the compensation structure in healthcare organizations during data collection, making it unavailable.

#### The explanation of the HRM modules [29]

1. Human resource planning is the starting point of HRM. It helps the organization forecast future personnel needs and their basic qualities, primarily through planning.
2. Recruitment and staffing, with HR planning as the input, is equivalent to the organization’s blood, nourishing the organization and solving the problem of staffing and staff matching.
3. Training and development, with the “education” theme.
4. Performance Management is at the heart of the six dimensions. It is also the primary input to the other dimensions.
5. Compensation management aims to motivate employees to solve the company’s problems.
6. Employee relations management aims to manage people and help the company form an effective cycle of rational human resource allocation.

#### Human resource planning

Human Resource Plan (HRP) stands for the implementation of the HR development strategy of the enterprise and the accomplishment of the enterprise’s goals, according to the changes in the internal and external environment and conditions of the enterprise, through the analysis and estimation of the future needs and supply of human resources and the use of scientific methods for organizational design, as well as the acquisition, allocation, utilization and maintenance of HR and other aspects of functional planning. HRP ensures that the organization has a balance of HR supply and demand at a needed time and in a required position, and achieves a reasonable allocation of HR and other resources to effectively motivate and develop of employees [34].

Decentralization health system, organizational culture/structure are high-frequency words in the clustering results related to “human resource management”. It is important to assess the extent to which decentralization can be used as a policy tool to improve national health systems. For policymakers and managers, based on relevant literature and research as well as country experience analysis, the experience of decentralization in relation to the organization and management of healthcare services is considered a forward-looking and pioneering concept capable of achieving optimal allocation of HR and other resources, in addition to the need to focus more on ex-ante and ex-post incentive development to deliver a 1 + 1 > 2 HRM effect [35]. HRP is the starting point and basis for all specific HRM activities. It directly affects the efficiency of the overall HRM of the enterprise. It is, therefore, taken as the primary job requirement for HR managers [36]. Organizational culture/structure significantly impacts the healthcare sector, such as excellence in healthcare delivery, ethical values, engagement, professionalism, cost of care, commitment to quality and strategic thinking, which are key cultural determinants of high-quality care delivery [37]. Therefore, as with other for-profit organizations, healthcare organizations must ensure that their organizational structure functions effectively to achieve their strategic goals. The organization formulates and implements HRM, an important task to achieve the development strategy goals.

#### Staff recruitment and allocation

Recruitment and staffing are the first steps in hospital HRM activities. Under the guidance of the organization’s human resources development plan, potential staff who meet the development conditions are attracted. Through the scientific selection of outstanding personnel, a platform with guaranteed treatment and development prospects is provided to ensure that the team of the healthcare organization is built solidly and meets the development needs. From the findings of this study, the keywords “workforce” and “workload” appear as high-frequency keywords in the co-occurrence analysis. Still, keywords related to traditional staff recruitment (e.g., analysis of recruitment needs, job analysis, competency analysis, recruitment procedures, and strategies) do not appear often. Recruitment and staffing are the prerequisites of human resources work. They bring a new dynamic source to healthcare organizations while complementing staff, making the organization full of vitality and vigor, facilitating organizational innovation and management innovation and helping improve the healthcare organization’s competitive advantage [38]. Recruitment and staffing, as a part of HR, directly impact the successful running of daily activities.

#### Training and development

Human resource training is an important component of quality and safety in the health care system. The keyword “education and training” shows a high frequency of co-occurrence in the clustering results of analysis, corresponding to the module “training and education”. However, it is connected to the keywords “human resource management” and “health policy”, and is in the same cluster with” public health”, “health care management”, and the distance between the lines and dots indicate that these topics are closely related, proving the importance of education and training in the HRM of health systems. Healthcare organizations (especially for non-professionals and caregivers) can improve the performance of their employees by enhancing their capabilities, knowledge and potential through learning and training, so that they can maximize their qualifications to match the demands of their work and advance their performance [39, 40].

#### Performance management

Performance management, the core of the six modules, is also featured in the clustering results. Although this is an important focus for HR professionals, few studies have explored the link between HRM and health sector performance [6], the results show “performance” and “motivation”. The effectiveness of performance management is an important component of HRM, which effectively improves the quality of care in healthcare organizations/institutions [6]. Focusing on the effectiveness of performance management is considered to be crucial. First, as an integral part of HRM within an organization, it can help the organization meet its goals. Second, ineffective approaches can lead to negative attitudes among employees (including clinicians, nursing staff, administrators, etc.) and adversely affect performance due to decreased satisfaction among employees and patients. Third, given the increasing quality and cost reduction pressures on healthcare organizations, conducting further research on performance management and effectiveness is critical [41]. However, it is clear from our results that healthcare organizations have recognized the importance of performance management and are pursuing “high performance”. Although the topic of performance management in HRM in healthcare is one of the research priorities, the number is lacking and more discussion on performance management should be suggested for future research.

#### Compensation management

Compensation is an important tool to motivate employees to work hard and to motivate them to work hard. The results of the database's bibliographic analysis show that no keywords directly involved compensation. This indicates that “compensation management” has not been considered a hot topic or a research issue over 30 years of available literature. To clarify the content of this module, we further searched the database of 718 articles with keywords, such as compensation, remuneration, salary, etc., and found that only 35 of them mentioned or discussed compensation, and some years (e.g., 2018, 2009) even had no relevant literature being published. However, issues such as fairness of compensation management and employee compensation satisfaction are still important issues of concern to business management academics [42, 43]. The actual situation is that it is difficult to conduct research on compensation management. Most organizations keep their employees’ compensation confidential, and when conducting research, HR managers avoid talking about their employees’ compensation or leave it vague, rendering it impossible for researchers to conduct further research.

Employee compensation is one factor that has the greatest impact on organizational performance. In the future, organizations should be encouraged to scientifically structure their compensation management and empower academic research to establish and implement fair compensation management systems based on empirical research while maintaining the privacy and security of organizational information.

#### Employee relations management

The connotation of employee relations management involves organizational culture and employee relations, as well as the coordination of the relationship between employers and employees. Healthcare organizations have complex structures with employees with varying skills, tasks or responsibilities, and such conflicts are often managed through the communication skills of administrative staff [44]. Although the keywords related to “employee relations management” did not occur in this study's analysis results, the six HRM modules are closely related. Therefore, this does not mean that no description of employee relations management was completely absent in the retrieved articles. It is clear that there is currently a lack of research on employee relations management in the healthcare field. Still, with the continuous development of the healthcare industry, it faces multiple challenges. If employee relations are not handled properly, healthcare organizations with social responsibility will face great public pressure, which will even affect the quality of healthcare services and performance, so it is especially important to strengthen the research on employee relations management.

## Limitation

This study inevitably has some limitations, the first of which arises from using quantitative methods to review documents in the field of HRM. The review relied on an analysis of the bibliographic data associated with the documents rather than a review of the research findings. The impact of the study was, therefore, limited to the general direction of developments in the field, rather than a synthesis of research findings. As a result, we may have missed some publications due to database bias. Second, most of the publications identified were in English and some articles relevant to other languages have not been included. Third, Since HRM exists in a wide range of industries and research areas, although researchers have set the screening criteria as detailed as possible, there may still be some literature that has not been detected.

## Conclusion

This study describes the current state and global trends in HRM research in healthcare. The United States has made significant contributions in this field, establishing itself as a global leader. It is foreseeable that more and more publications will be published in the coming years, which indicates that HRM research in healthcare is booming. The analysis results of this study echoed the modules of HRM. It can be seen that in the current HRM research, many topics have been of interest. However, the focus and hotspots of the research are scattered, and there is presently no systematic research on the content of HRM in healthcare.

## Data Availability

All data and materials generated or analysed during this study are included in this published article.
